# Effects of the nitrate and ammonium ratio on plant characteristics and *Erythropalum scandens* Bl. substrates

**DOI:** 10.1371/journal.pone.0289659

**Published:** 2023-08-04

**Authors:** Daocheng Ma, Weichao Teng, Biao Yi, Yongzhi Lin, Yuanyuan Pan, Linghui Wang

**Affiliations:** College of Forestry, Guangxi University, University Road, Nanning, Guangxi Zhuang Autonomous Region, 530004, China; Universidad Autónoma Agraria Antonio Narro, MEXICO

## Abstract

*Erythropalum scandens* Bl. is a woody vegetable with high nitrogen demand that inhabits southern China. Ammonium and nitrate are the two main forms of inorganic nitrogen that plants directly absorb. A pot experiment was performed to determine the growth, physiological responses, and preferences of 12-month-old *E*. *scandens* seedlings for ammonium and nitrate. Aboveground and underground growth indexes, biomass, physiological and biochemical indexes (chlorophyll [Chl], soluble sugar, soluble protein and free proline contents), and substrate pH and nitrogen contents were determined under different nitrate and ammonium ratios (0 NO_3_^-^: 100 NH_4_^+^, 25 NO_3_^-^: 75 NH_4_^+^, 50 NO_3_^-^: 50 NH_4_^+^, 75 NO_3_^-^: 25 NH_4_^+^, and 100 NO_3_^-^: 0 NH_4_^+^), and the control (0 NO_3_^-^: 0 NH_4_^+^). The results showed that ammonium and nitrate improved the growth and physiological status of *E*. *scandens* seedlings in most of the treatments compared to the control. The aboveground growth status and biomass accumulation of *E*. *scandens* seedlings were significantly better under the 0 NO_3_^-^: 100 NH_4_^+^ treatment during fertilization compared with all other treatments. However, the growth status of the underground parts was not significantly different among treatments. Significant differences in osmoregulator content, except for soluble sugars, and Chl content were observed. Soluble sugars and soluble proteins were highest under the 0 NO_3_^-^: 100 NH_4_^+^ treatment at the end of fertilization (day 175). However, free proline accumulated during fertilization and the increase in NO_3_^-^ indicated that excessive use of NO_3_^-^ had a negative effect on the *E*. *scandens* seedlings. The order of accumulating nitrogen content was leaves > roots > stems. The highest N accumulation occurred in the aboveground parts under the 0 NO_3_^-^: 100 NH_4_^+^ treatment, whereas the highest N accumulation occurred in the underground parts under the 50 NO_3_^-^: 50 NH_4_^+^ treatment. Substrate pH increased at the end of fertilization (day 175) compared with the middle stage (day 75), while total nitrogen, ammonium, and nitrate were highly significantly different among the treatments. Total nitrogen and NH_4_^+^ content were the highest under the 0 NO_3_^-^: 100 NH_4_^+^ treatment, while NO_3_^-^ content was the highest under the 100 NO_3_^-^: 0 NH_4_^+^ treatment. In conclusion, 12-month-old *E*. *scandens* seedlings grew best, and had better physiological conditions in NH_4_^+^ than NO_3_^-^. The 0 NO_3_^-^:100 NH_4_^+^ treatment (ammonium chloride 3.82 g/plant) resulted in the best growth and physiological conditions. Most of the growth and physiological indexes were inhibited with the increase in nitrate.

## 1 Introduction

*Erythropalum scandens* Bl. (family Olacaceae) is a woody leafy vegetable inhabiting south and southwest China, Vietnam, and other Southeast Asian countries. Its tender leaves and stems have a distinct taste and smell. Due to its high nutritional value and pleasing taste, it is often picked and eaten by farmers [1]. *E*. *scandens* has a large planting area in Guangxi, particularly in Daxin County (about 67 hectares), as it is an important national plant resource in western and southern China. The tender stems and leaves are sold as a wild vegetable for 40–100 CNY/kg. The estimated production output is 66.5 kg/ha/year, with an output value of > 2,660–6,650 CNY/ha/year.

*Erythropalum scandens* also functions in soil and water conservation [2]. It is shade-tolerant in rain forests and limestone mountainous areas, and is a widely distributed “interlayer plant” in Xishuangbanna, Yunnan, and other areas of south and southwest China [3]. Although it grows well under natural conditions, the yield of *E*. *scandens* is limited in the natural environment. *E*. *scandens* and its germplasm resources have been seriously damaged in the wild. Therefore, how to improve the yield of *E*. *scandens* under an artificial cultivation environment, meet the growing demand therefor, and reduce destruction of the wild resource are top priorities. The current study showed that fertilization greatly improved the yield and quality of *E*. *scandens*. In addition, Guo [4] reported that a combination of chicken manure (0.8 kg/plant) and cow manure (1.2 kg/plant) was the best method to increase the yield of 24-month-old *E*. *scandens* cutting seedlings; their edible parts accumulated carbohydrates, amino acids and other nutrients, as well as medicinal components. The average bud and leaf yield was 20.58 g after 7 months of fertilization. Ma et al. [5,6] showed that the growth and physiological conditions of 18-month-old *E*. *scandens* seedlings were optimal under a 2.14 g/plant urea + 4.44 g/plant superphosphate + 0.67–1.33 g/plant potassium chloride treatment. The number of new leaves per plant under the optimal fertilization treatment was 41.5, and the length of new branches was 147.52 cm. According to these studies, nitrogen plays a very important role in leaf growth and stem development of *E*. *scandens*. However, previous studies used organic fertilizers and urea as N sources. The components of organic fertilizers are complex, and it is difficult to determine which N-containing substances best promote the growth and development of *E*. *scandens*, as urea is an amide nitrogen [CO(NH_2_)_2_-N] that is difficult for some plants to absorb. In contrast, NH_4_^+^-N and NO_3_^—^N are two types of inorganic nitrogen that can be directly absorbed by plants [7,8]. Therefore, it is necessary to understand NH_4_^+^-N and NO_3_^—^N absorption and utilization by *E*. *scandens*.

NH_4_^+^-N and NO_3_^—^N affect plants in different ways. Some studies have reported that low concentrations of NH_4_^+^-N and NO_3_^—^N have similar effects on plants, but many plants have different preferences and tolerances under high NH_4_^+^-N and NO_3_^—^N concentrations [9–11]. In addition, combined application of NH_4_^+^-N and NO_3_^—^N plays an important role in chlorophyll (Chl) synthesis and trace element absorption in plants. For example, applying ammonium nitrate is more conducive to the accumulation of plant biomass than applying urea and calcium nitrate [12]. Studies have shown that some plants prefer pure NH_4_^+^-N, some prefer pure NO_3_^—^N, some prefer an equal amount of each, and some prefer a combination; these are the most common forms of NH_4_^+^-N and NO_3_^—^N for plants [13]. Different plant species have distinct preferences for the various nitrogen forms at different growth stages. For example, some conifers, such as *Picea glauca* and *Pinus sylvestris*, prefer NH_4_^+^-N [14]. Sugarcane (*Saccharum* spp.) has high nitrogen utilization efficiency and the best leaf and root growth conditions after a single NO_3_^—^N application [15]. Coffee and oil tea prefer a 50:50 NH_4_^+^-N and NO_3_^—^N ratio [16,17], as do *Cyrtanthus guthrieae* [18] and *Beta vulgaris* [19]. The most variable combination is that of NH_4_^+^-N and NO_3_^—^N. For example, lettuce and potatoes perform best at a 75:25 nitrate/ammonium ratio [20,21]. Low NH_4_^+^-N/NO_3_^—^N promotes the growth of pine and pepper, and increases the leaf biomass and accumulation of N, P, and K [22,23]. When NH_4_^+^-N/NO_3_-N = 25:75, the contents of sugar, protein, total phenol, flavonoid, vitamin C, and other nutrients of pepper increase significantly. Legumes prefer NO_3_^—^N during the entire growth process, while gramineous plants prefer NH_4_^+^-N at the early growth stage, but subsequently favor NO_3_^—^N [24]. Biomass accumulation, root growth, and phosphorus uptake are promoted in the five-leaf stage after CaHPO_4_ application [25].

The effects of N form on plants depend on plant and environmental factors [26,27]. For example, a 8 mmol L^−1^ Ca NO_3_^—^N treatment resulted in the highest nutritional value and quality of tomato fruits [28]. A 4:11 NH_4_^+^-N/NO_3_^—^N ratio and 5.6 mmol/L potassium, and 5:10 NH_4_^+^-N/NO_3_^—^N and 6.0 mmol/L potassium, improved the growth of *Raphanus sativus* [29]. *Coix lacryma-jobi* L. prefers NO_3_^—^N nutrition under neutral and alkaline conditions [30]. *Panax notoginseng* can survive in a shady environment for a long time in very wet soil. In moist soil, the number of microorganisms controlling nitrification decreases due to hypoxia, so NH_4_^+^-N accumulates in large quantities and is difficult to convert to other forms [31]. In addition, NO_3_^—^N is better-absorbed than NH_4_^+^-N in deep soil with low soil nitrogen content [32]. In summary, different plants have distinct preferences for NH_4_^+^-N or NO_3_^—^N, depending on the specific situation. A reasonable NH_4_^+^-N and NO_3_^—^N ratio is important to regulate growth and physiology.

Based on a previous fertilization study, we conducted pot experiments to determine the effects of different NH_4_^+^-N and NO_3_^—^N ratios on the growth, physiology, and substrates properties of *E*. *scandens* seedlings. Specifically, we evaluated aboveground and underground growth indexes, biomass, physiological and biochemical indexes (Chl, soluble sugar, soluble protein, and free proline contents) and the pH and nitrogen content of the substrate to assess the preference and adaptability of different nitrogen forms and lay a foundation for the application of nitrogen fertilizer for *E*. *scandens*.

## 2 Materials and methods

### 2.1 Ethics statement

*E*. *scandens* Bl. is an edible woody vegetable in South and Southwest China. It is not included on the IUCN Red List of Threatened Species. The pot experiment was approved by the College of Forestry, Guangxi University. The plants were transplanted and maintained following ethical guidelines to ensure normal growth and regulation of *E*. *scandens* seedlings (the information of seedlings growth situation was as follow).

### 2.2 Test site and materials

**1) Test site:** The experiment was conducted in a greenhouse (108°22’E, 22°48’N) at the teaching nursery of the College of Forestry, Guangxi University, which has a subtropical monsoon climate (mild and humid, with an average annual temperature of 21.6°C and rainfall of 1,304 mm). The highest temperature recorded was 39.5°C. The greenhouse was covered with a layer of plastic film and a layer of black shading net. The light in the greenhouse was 70% that of the natural conditions.

**2) Seedlings:** Twelve-month-old *E*. *scandens* seedlings were provided by the Nanning Arboretum in Guangxi, Daxin County, China. Mature seeds were selected in 2021 after sowing. When the buds reached 5 cm, seeds with similar growth were selected and transplanted into non-woven planting bags (9 cm in height and 6 cm in diameter) with red soil, for cultivation and to restore their growth potential. The red soil (understory soil from a eucalyptus plantation) was provided by Nanning Arboretum in Guangxi. Seedlings with similar growth were selected for the pot experiment. The baseline growth indexes were as follows: plant height, 21.74 ± 5.58 cm; and ground diameter, 5.86 ± 1.10 mm. The initial nitrogen content of a whole seedling was 14.74 g/kg. No significant group differences were observed in the initial growth indexes before the experiment (*P* > 0.05).

**3) Substrates:** A mixture of yellow soil, fine sand, perlite, and peat was evenly mixed at a volume ratio of 6:2:1. The yellow soil and fine sand were obtained from the teaching nursery of the College of Forestry, and the perlite and peat were provided by Nanning Guiyuxin Agricultural Technology Co. Ltd. The basic physicochemical properties of the substrate were as follows: pH, 6.44; total nitrogen, 2.021 g/kg; total phosphorus, 1.305 g/kg; total potassium, 7.469 g/kg; ammonium-nitrogen, 20.128 mg/kg; and nitrate-nitrogen, 39.832 mg/kg. The diameter and height of the round plastic pots were 15.30 and 14.90 cm, respectively, and one seedling was planted per pot. A round plastic tray was placed under the basin to prevent fertilizer leakage.

### 2.3 Experimental design

All seedlings were transplanted in early March 2022 and separated into groups. According to the results of previous experiments [5,6] and the baseline value of substrate nitrogen in this experiment, the total amount of nitrogen added was 1 g/plant. In accordance with Hua et al. [33], 1 g of pure nitrogen was divided into five combinations of nitrate and ammonium: 0 NO_3_^-^: 100 NH_4_^+^, 25 NO_3_^-^: 75 NH_4_^+^, 50 NO_3_^-^: 50 NH_4_^+^, 75 NO_3_^-^: 25 NH_4_^+^, and 100 NO_3_^-^: 0 NH_4_^+^. A treatment without fertilization was used as the control (Table 1).

**Table 1:** Description of the treatments.

Treatment	Nitrate-nitrogen (NO_3_^-^-N)		Ammonium-nitrogen (NH_4_^+^-N)	
	Pure nitrogen content (g/plant)	Fertilizer amount (g/plant)	Pure nitrogen content (g/plant)	Fertilizer amount (g/plant)
Control	0	0	0	0
0 NO_3_^-^: 100 NH_4_^+^	0	0	1.00	3.82
25 NO_3_^-^: 75 NH_4_^+^	0.25	2.11	0.75	2.87
50 NO_3_^-^: 50 NH_4_^+^	0.50	4.22	0.50	1.91
75 NO_3_^-^: 25 NH_4_^+^	0.75	6.32	0.25	0.96
100 NO_3_^-^: 0 NH_4_^+^	1.00	8.43	0	0

NH_4_Cl (pure nitrogen content, 26.17%) and Ca (NO_3_) _2_·4H_2_O (pure nitrogen content, 11.86%) were used as the NH_4_^+^-N and NO_3_^—^N sources, respectively. Each treatment was set up with 18 biological replicates (one basin per replicate), and 9 were used for destructive sampling (3 replicates each for biomass, the physiological indexes, and the underground indexes). During this study (April–September 2022), the fertilizer was added by irrigation. According to the nutrient absorption and growth status of *E*. *scandens* seedlings in our previous experiment, the fertilizer application rates were 20%, 25%, 30%, 15%, and 10% of the total amount on 9 April, 14 May, 18 June, 23 July, and 27 August, respectively. In total, 200 mL of formula solution was poured into each pot on each occasion, with an application interval of 35 days. All pots were placed randomly during fertilization and the substrate was kept moist at 60% of field capacity. All seedlings were irrigated according to the weather conditions. Seedlings were irrigated every other day from March to mid-April, but seedlings were irrigated once a day due to the hot weather from late April to September until the end of the experiment. The pots were checked daily for weeds, which were pulled when discovered. The growth and physiological indexes were measured during and after the experiment.

### 2.4 Indexes determination

#### 2.4.1 Growth

*2*.*4*.*1*.*1 Aboveground growth*. The aboveground growth indexes were measured 30 days after each fertilizer application. The length of the new branches and internodes was measured with a steel tape to within 0.01 cm. The ground diameter and thickness of new branches were measured with electronic Vernier calipers to within 0.01 mm. The numbers of buds, new leaves, and new/basal branches were recorded based on visual inspection. All seedlings were measured.

*2*.*4*.*1*.*2 Underground growth*. On day 175 after the first fertilizer application, three healthy seedlings were randomly selected from each treatment to determine the root growth indexes. The roots were dug up and carefully washed with tap water and deionized water. The roots were divided into different groups using scissors and scanned with the Epson Expression 10000XL system. The WinRHIZO root analysis system was used to determine all indexes, including total root length, total root surface area, total root projection area, total root volume, and the average diameter to within 0.001 cm, 0.001 cm^2^, 0.001 cm^2^, 0.001 cm^3^, and 0.001 mm, respectively. Total root length, total root surface area, total root projection area, and total root volume were determined to group the roots. The mean root diameter was averaged after measuring the diameter of all fibrous roots. Three healthy seedlings were randomly selected to measure the underground growth indexes.

*2*.*4*.*1*.*3 Biomass accumulation*. On day 175 after the first fertilizer application, three healthy seedlings were randomly selected from each treatment. The plants were washed in tap and deionized water, and then wiped clean. Each part was wrapped in a clean brown paper envelope and placed in an oven. After drying the plants at 105°C for 30 min, the plants were dried at 75°C to constant weight and the dry weight was determined to within 0.01 g.

#### 2.4.2 Physiological and biochemical indexes

The physiological and biochemical indexes of mature functional leaves of the *E*. *scandens* seedlings were measured on days 75 and 175 after the first fertilization. The second to fifth mature functional leaves were collected from the top bud of the upper branch of the plant from three seedlings with similar growth status.

*2*.*4*.*2*.*1 Chlorophyll content*.About 0.15 g of fresh leaf tissue (without the leaf vein was cut into pieces and added to 5 mL of extraction solution (acetone: absolute ethanol: deionized water = 4.5: 4.5: 1 [v/v/v]) and placed in the dark for 48 h, with intermittent oscillations for 12 h until the leaf was completely white. Absorption values of the samples were recorded at 663 and 645 nm using an ultraviolet spectrophotometer (UV-2450; Shimadzu, Tokyo, Japan) [34].

1) Chl *a* = (12.70OD_663_ − 2.69OD_645_) × $\frac{V}{1000W}$

2) Chl *b* = (22.90OD_645_ − 4.68OD_663_) × $\frac{V}{1000W}$

3) Chl *a + b* = (20.20OD_645_ + 8.02OD_663_) × $\frac{V}{1000W}$

Where V represents the volume of extraction liquid (5 mL) and W represents fresh leaf sample weight.

*2*.*4*.*2*.*2 Soluble sugar content*.About 0.15 g of dried leaf sample was added to 15 mL of deionized water and extracted in a boiling water bath for 30 min; this process was repeated twice. Then, the supernatants of the two extracts were combined into a 50 mL volumetric flask, and the volume of deionized water was fixed according to the soluble sugar extract. The ethyl acetate reagent of anthrone was obtained by dissolving 0.5 g anthrone in 100 mL of ethyl acetate. After the sugar extract was fully cooled, 0.5 mL was added to a 50 mL test tube; 0.5 mL anthrone ethyl acetate reagent and 5 mL concentrated sulfuric acid were then added successively and the mixture was placed in a boiling water bath for 1 min. The mixture turned completely blue, and was cooled and analyzed by a colorimeter at a wavelength of 630 nm. Soluble sugar content was determined by colorimetry and a standard curve. A 100 μg/mL standard solution was prepared with sucrose and deionized water, and then diluted with deionized water into working solutions with concentrations of 0–100 μg. The INFINITE M200 PRO instrument (Tecan, Männedorf, Switzerland) was used for colorimetry [35].

*2*.*4*.*2*.*3 Soluble protein content*.About 0.15 g of fresh leaf tissue (without the leaf vein) was ground into a homogenate in cold 0.2 M phosphate buffer (pH 7.0). The homogenate was centrifuged twice at 4,000 rpm for 10 min at 4°C and then homogenized with 10 mL of deionized water. The supernatant was collected as the enzyme extract. A 0.1 mL aliquot of the protein extract, 0.9 mL of deionized water, and 5 mL of Coomassie Brilliant Blue (CBB) solution (500 mL, including 0.5 g CBB, 50 mL of 85% phosphoric acid, and 25 mL 95% ethanol) were added successively to a 25 mL test tube. After fully mixing, the liquid was left for 5 min and colorimetry was performed at 595 nm. Soluble sugar content was determined by colorimetry and a standard curve. A 1,000 μg/mL standard solution was prepared with bovine serum protein and deionized water, and then diluted with deionized water into working solutions with concentrations of 0–1,000 μg/mL. The INFINITE M200 PRO instrument was used for colorimetry [36].

*2*.*4*.*2*.*4 Free proline content*.About 0.15 g of fresh leaf tissue (without the leaf vein) was homogenized in 1.5 mL of 3% sulfosalicylic acid in a 5 mL tube. The mixture was placed in a boiling water bath for 10 min followed by centrifugation at 10,000 rpm for 5 min. Then, 300 μL of the supernatant was added to separate tubes, and 2 mL each of glacial acetic acid and acid ninhydrin (1.25 g ninhydrin warmed in 30 ml of glacial acetic acid and 20 mL of 6 mol/L phosphoric acid until dissolved) was added and held for 1 h in the boiling water bath. The tubes were removed from the water bath and immediately stored in ice until the reaction was completed. Then, 5 mL of toluene was added and mixed vigorously with the reaction mixture for 10–30 s. The organic phase was measured at a wavelength of 520 nm. Toluene was used as the blank (control). The proline concentrations of the different samples were determined from a standard curve. Proline reagent and deionized water were used to make a 10 μg/mL standard solution, and deionized water was then diluted into the working solution to prepare concentrations of 0–20 μg. After being heated and extracted with toluene, the INFINITE M200 PRO instrument was used for colorimetric analysis of the upper phase using the same method [37].

Three biological replicates were selected for each physiological index measured (one plant per replicate).

#### 2.4.3 Plant nitrogen content

Three seedlings were randomly selected from the plants treated in each treatment on days 70 and 175 after the first fertilization. The second to fifth mature functional leaves were taken from the top bud of the upper branch, and the leaves were dried and boiled in H_2_SO_4_-H_2_O_2_. The drying method was the same as that used for determining biomass. Then, to prepare the standard curve, a 1,000 mg/L standard solution was prepared with (NH_4_)_2_SO_4_ analytical reagent and deionized water to concentrations of 0–40 mg/L. The total nitrogen content of the standard curve and samples was determined using a continuous flow analyzer (AA3; Bran + Luebbe, Hamburg, Germany), with three biological replicates per treatment.

#### 2.4.4 Substrate pH and nitrogen content

1) pH: Substrate samples from each treatment were collected on days 70 and 175 after the first fertilization. The substrate from each treatment was air-dried and passed through a 2 mm sieve. Then, two 5 g samples were weighed and soaked in 25 mL of CO_2_ with deionized water and 1 mol/L KCl (aq), respectively. The samples were extracted by intermittent stirring for 30 min and the pH of the upper liquid was measured with a pH meter (FE28; Mettler Toledo, Basel, Switzerland). The neutral and alkaline substrates (pH > 6.5) were extracted with deionized water and the acidic substrate (pH < 6.5) was extracted with 1 mol/L KCl (aq).

2) Nitrogen content: Substrate samples from each treatment were collected on days 70 and 175 after the first fertilization, respectively, to determine the contents of total nitrogen, NH_4_^+^, and NO_3_^-^. After removing 2 cm of top substrate, about 5 g of substrate was collected from each pot and mixed evenly as the sample for each treatment. Then, the mixed substrate samples were separated into two parts. The first part was air-dried and sieved through 100 mesh to determine the total nitrogen content. The second part was kept fresh and sieved through 18 mesh to determine the NH_4_^+^-N and NO_3_-N contents. Total nitrogen content was determined with H_2_SO_4_-CuSO_4_+K_2_SO_4_ by boiling and using the AA3 continuous flow analyzer. NH_4_^+^ and NO_3_^-^ were extracted with 0.1 mol/L CaCl_2_ (aq) (substrate-water ratio, 1:5) and then determined by the AA3 continuous flow analyzer. The standard curve was prepared as described above.

### 2.5 Statistical analyses

Microsoft Excel 2016 (Microsoft Corp., Redmond, WA, USA) was used to sort the index data. All statistical analyses were performed using SPSS software (version 18.0; IBM Corp., Armonk, NY, USA). Duncan’s new complex range method (*P* = 0.05) was used for multiple comparisons. Data were graphed with Microsoft Excel 2016. A P-value < 0.05 was considered significant.

## 3 Results

### 3.1 Growth indexes

**1) Aboveground growth:** Except for the number of new buds, new branches, basal branches, and the increase in ground diameter, significant or extremely significant differences were detected between the treatments (Table 2 and S1 Table in S1 File). Except for the increase in ground diameter, the other indexes all reached their maxima under the 0 NO_3_^-^:100 NH_4_^+^ treatment: 5.50 for new buds, 21.06 for new leaves, 3.94 for new branches, 0.50 for basal branches, 4.44 for the sum of branches, 66.42 cm for the total length of new branches, 19.17 for the number of new internodes, 3.51 cm for the mean length of internodes, and 1.70 mm for new branch thickness. Meanwhile, ground diameter reached its maximum under the 50 NO_3_^-^:50 NH_4_^+^ treatment (2.70 mm). In general, as more NO_3_^-^ was added, germination and the leaf and branch growth of the *E*. *scandens* seedlings were gradually inhibited.

**Table 2:** Aboveground growth status of *E*. *scandens* seedlings under the different fertilization treatments.

**Treatment**		**Number of new buds**		**Number of new leaves**		**Number of new branches**		**Number of basal branches**		**Sum of branches**
Control		4.67 ± 2.59 a		11.11 ± 6.95 c		2.78 ± 2.16 a		0.11 ± 0.47 a		2.89 ± 2.22 b
0 NO_3_^-^: 100 NH_4_^+^		5.50 ± 2.31 a		21.06 ± 8.79 a		3.94 ± 1.70 a		0.50 ± 0.99 a		4.44 ± 2.20 a
25 NO_3_^-^: 75 NH_4_^+^		4.61 ± 1.88 a		16.00 ± 5.97 bc		2.94 ± 1.11 a		0.28 ± 0.57 a		3.22 ± 1.22 b
50 NO_3_^-^: 50 NH_4_^+^		4.22 ± 1.96 a		16.61 ± 6.70 ab		2.72 ± 0.96 a		0.28 ± 0.57 a		3.00 ± 0.84 b
75 NO_3_^-^: 25 NH_4_^+^		4.28 ± 2.30 a		14.33 ± 5.69 bc		2.83 ± 1.04 a		0.00 ± 0.00 a		2.83 ± 1.04 b
100 NO_3_^-^: 0 NH_4_^+^		4.17 ± 1.62 a		15.50 ± 6.92 bc		2.89 ± 1.23 a		0.39 ± 0.85 a		3.28 ± 1.49 b
**Treatment**	**Total length of new branches (cm)**		**Number of new internodes**		**Mean length of internode (cm)**		**Ground diameter increment (Δmm)**		**New branch thickness (Δmm)**	
Control	21.79 ± 14.34 c		9.83 ± 5.90 c		2.12 ± 1.04 b		1.94 ± 0.65 a		1.09 ± 0.68 b	
0 NO_3_^-^: 100 NH_4_^+^	66.42 ± 39.42 a		19.17 ± 8.35 a		3.51 ± 1.65 a		1.95 ± 0.88 a		1.70 ± 0.55 a	
25 NO_3_^-^: 75 NH_4_^+^	43.17 ± 25.24 b		14.67 ± 6.33 b		2.87 ± 1.21 ab		2.48 ± 0.89 a		1.52 ± 0.42 a	
50 NO_3_^-^: 50 NH_4_^+^	44.80 ± 26.32 b		14.22 ± 5.92 bc		2.94 ± 1.21 ab		2.70 ± 0.93 a		1.51 ± 0.45 a	
75 NO_3_^-^: 25 NH_4_^+^	31.73 ± 22.84 bc		13.61 ± 5.75 bc		2.23 ± 1.09 b		2.26 ± 0.90 a		1.57 ± 0.60 a	
100 NO_3_^-^: 0 NH_4_^+^	33.30 ± 22.70 bc		13.78 ± 6.64 bc		2.25 ± 1.13 b		2.08 ± 1.03 a		1.46 ± 0.52 a	

Note: Values are mean ± standard deviation (n = 18); different lowercase letters indicate a significant difference between treatments (P < 0.05).

**2) Underground growth:** Except for total root length, no significant differences were detected among treatments (Table 3 and S2 Table in S1 File). Under the 75 NO_3_^-^: 25 NH_4_^+^ treatment, total root surface area, total root projection area, and total root volume reached their maxima (129.00 cm^2^, 41.06 cm^2^, and 2.64 cm^3^, respectively). Total root length reached its maximum under the 50 NO_3_^-^: 50 NH_4_^+^ treatment (576.50 cm). Mean root diameter reached the maximum under the control (1.20 mm). All indexes first increased and then decreased with the increase in the amount of NO_3_^-^ added, although some differences were observed between indexes. Adding an appropriate amount of NO_3_^-^ promoted root growth of *E*. *scandens*, but root growth was slightly inhibited under the 100 NO_3_^-^: 0 NH_4_^+^ treatment.

**Table 3:** Effects of different nitrate and ammonium ratios on *E*. *scandens* root growth.

Treatment	Total root length (cm)	Total root surface area (cm^2^)	Total root projection area (cm^2^)	Total root volume (cm^3^)	Mean root diameter (mm)
Control	167.23 ± 18.86 b	75.48 ± 59.11 a	24.03 ± 18.82 a	1.48 ± 0.82 a	1.20 ± 0.53 a
0 NO_3_^-^:100 NH_4_^+^	576.50 ± 25.56 a	100.06 ± 11.74 a	31.85 ± 3.74 a	1.57 ± 0.67 a	0.65 ± 0.26 a
25 NO_3_^-^:75 NH_4_^+^	476.04 ± 95.91 a	106.95 ± 10.77 a	34.04 ± 3.43 a	1.99 ± 0.48 a	0.75 ± 0.16 a
50 NO_3_^-^:50 NH_4_^+^	673.18 ± 75.04 a	75.48 ± 6.05 a	24.03 ± 1.93 a	1.26 ± 0.80 a	0.41 ± 0.03 a
75 NO_3_^-^:25 NH_4_^+^	588.01 ± 236.52 a	129.00 ± 58.23 a	41.06 ± 18.53 a	2.64 ± 1.67 a	0.93 ± 0.30 a
100 NO_3_^-^:0 NH_4_^+^	576.38 ± 265.77 a	91.07 ± 29.87 a	29.02 ± 9.46 a	1.26 ± 0.59 a	0.55 ± 0.17 a

Note: Values are mean ± standard deviation (n = 18); different lowercase letters indicate a significant difference between treatments (P < 0.05).

**Fig 1:**
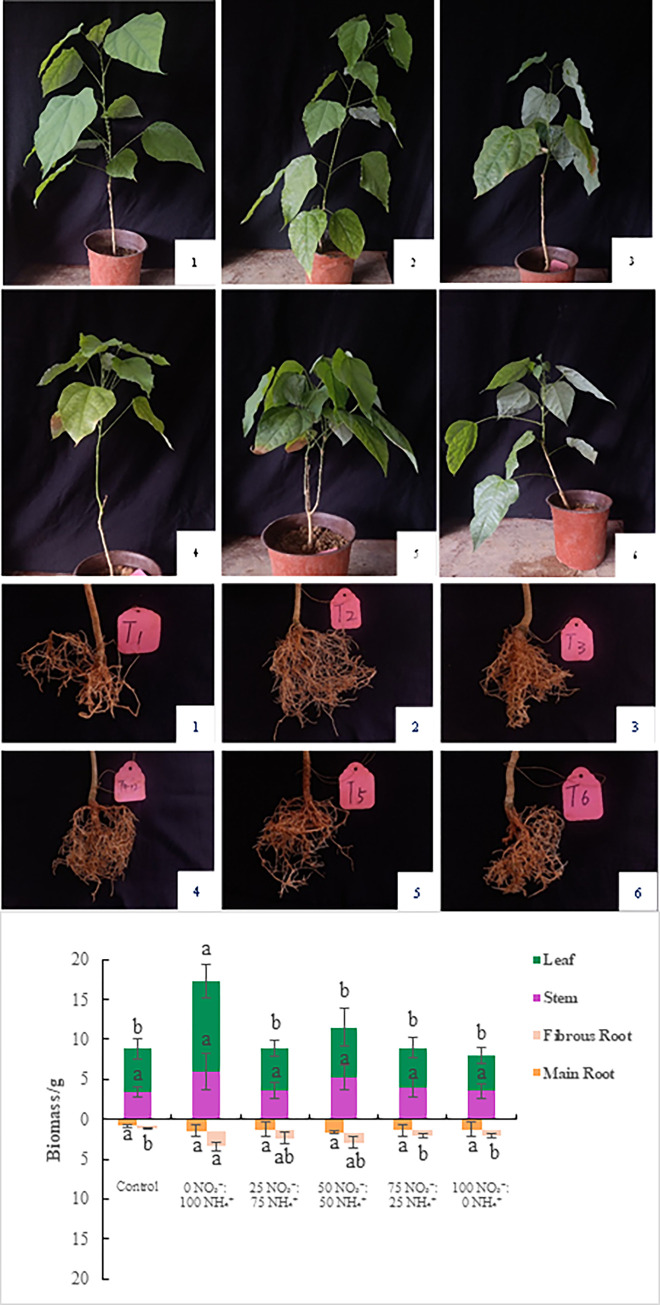
Biomass and growth status of *E*. *scandens* seedlings under the different fertilization treatments. Different lowercase letters indicate a significant difference between treatments (*P* < 0.05). Subscripts 1–6 in the figure represent the different treatments (1 = control, 2 = 0 NO_3_^—^:100 NH_4_^+^, 3 = 25 NO_3_^—^:75 NH_4_^+^, 4 = 50 NO_3_^—^:50 NH_4_^+^, 5 = 75 NO_3_^—^:25 NH_4_^+^ and 6 = 100 NO_3_^—^:0 NH_4_^+^.

**3) Biomass:** Unlike the main root and stem biomass, the fibrous root and leaf biomasses were significantly different among treatments (Fig 1 and S3 Table in S1 File). The main root biomass reached its maximum (2.02 g) under the 50 NO_3_^-^: 50 NH_4_^+^ treatment. The remaining parts reached their maxima under the 0 NO_3_^-^:100 NH_4_^+^ treatment: 2.40 g for fibrous root biomass, 4.23 g for total root biomass, 6.02 g for stem biomass, 11.30 g for leaf biomass, and 21.54 g for total biomass. With the increase in amount of NO_3_^-^ added, biomass increased first and then decreased, indicating that adding the appropriate amount of NO_3_^-^ promoted biomass accumulation in the main root, while the biomass of the fibrous root, stems, and leaves were greatest under the 0 NO_3_^-^:100 NH_4_^+^ treatment. Excessive NO_3_^-^ was not conducive to the growth or biomass accumulation of *E*. *scandens* seedlings.

**4) Growth status:** We observed wide variations in the aboveground and underground morphological status of *E*. *scandens* during the 175 days after the first fertilization treatment (Fig 1). The growth of the seedlings was gradually inhibited as NO_3_^-^ was increased, and the number of new leaves decreased. Root growth under the fertilization treatments was stronger than that in the control, but root morphology was not significantly different among the fertilization treatments, indicating that excessive use of NO_3_^-^ had adverse effects on *E*. *scandens* root growth.

### 3.2 Physiological characteristics

#### 3.2.1 Chlorophyll content

**Fig 2:**
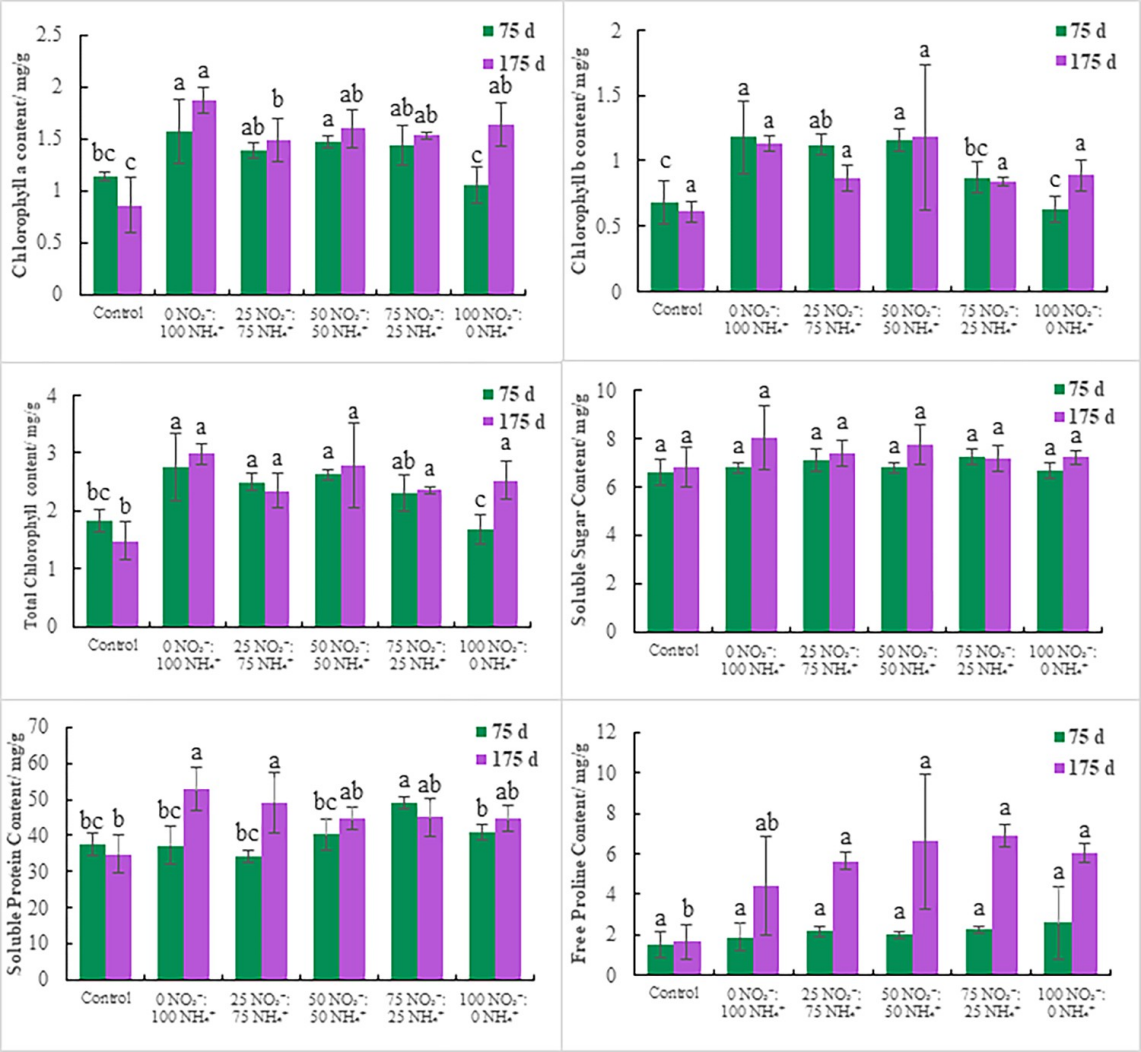
Physiological response of *E*. *scandens* seedlings under the different fertilization treatments. Different lowercase letters indicate a significant difference between the treatments (*P* < 0.05).

Chl *a* content at the end of the experiment (day 175) was higher than on day 75. Chl *b* and total Chl contents in most treatments were higher on day 175 than day 75. The Chl content in the control was lower on day 175 than day 75 (Fig 2). On day 75, significant differences in Chl *a*, Chl *b*, and total Chl content were observed among treatments (*P* < 0.05). No significant difference in Chl *b* content was detected on day 175 (*P* > 0.05), but there were significant differences in Chl *a* and total Chl content (*P* < 0.05) (S4 Table in S1 File). Chl *a*, Chl *b*, and total Chl content were highest on day 75 under the 0 NO_3_^-^:100 NH_4_^+^ treatment, reaching 1.57, 1.18, and 2.75 mg/g, respectively. Chl *a* and total Chl content were the highest on day 175 under the 0 NO_3_^-^:100 NH_4_^+^ treatment, while Chl *b* content was highest under the 50 NO_3_^-^:50 NH_4_^+^ treatment (1.87, 2.99, and 1.18 mg/g, respectively). The Chl content increased and then decreased as NO_3_^-^ increased. *E*. *scandens* Chl content was highest under the 0 NO_3_^-^:100 NH_4_^+^ treatment, and adding excessive NO_3_^-^ had negative effects on Chl synthesis and accumulation.

#### 3.2.2 Osmoregulator contents

Soluble sugar and soluble protein contents on day 175 after the first fertilization were higher than on day 75 in most treatments, while free proline content was higher on day 175 than day 75. In the control, soluble protein content was lower on day 175 than day 75, whereas soluble sugar and proline contents were higher than on day 75 (Fig 2). Soluble sugar and free proline contents were not significantly different among the treatments (*P* > 0.05) on day 75, whereas soluble protein content was significantly different (*P* < 0.05). No significant difference in soluble sugar content (*P* > 0.05) was detected among treatments on day 175, but there were significant differences in soluble protein and free proline contents (*P* < 0.05) (S5 Table in S1 File). Soluble sugar, soluble protein, and free proline contents reached their maxima on day 75 in the 75 NO_3_^-^:25 NH_4_^+^, 75 NO_3_^-^:25 NH_4_^+^, and 100 NO_3_^-^:0 NH_4_^+^ treatments (7.27, 48.91, and 2.59 mg/g, respectively). The maximum values of the three osmoregulators on day 175 were 8.05, 52.76, and 6.91 mg/g in the 0 NO_3_^-^:100 NH_4_^+^, 0 NO_3_^-^:100 NH_4_^+^, and 75 NO_3_^-^:25 NH_4_^+^, treatments, respectively. As NO_3_^-^ increased, soluble sugar and soluble protein contents increased first and then decreased, while free proline content accumulated to different degrees. The results show that using an appropriate amount of NO_3_^-^ promoted the synthesis of osmoregulators in the leaves of *E*. *scandens*, but excessive use of NO_3_^-^ resulted in the accumulation of free proline, which is not conducive to plant growth.

### 3.3 Plant nitrogen content

**Fig 3:**
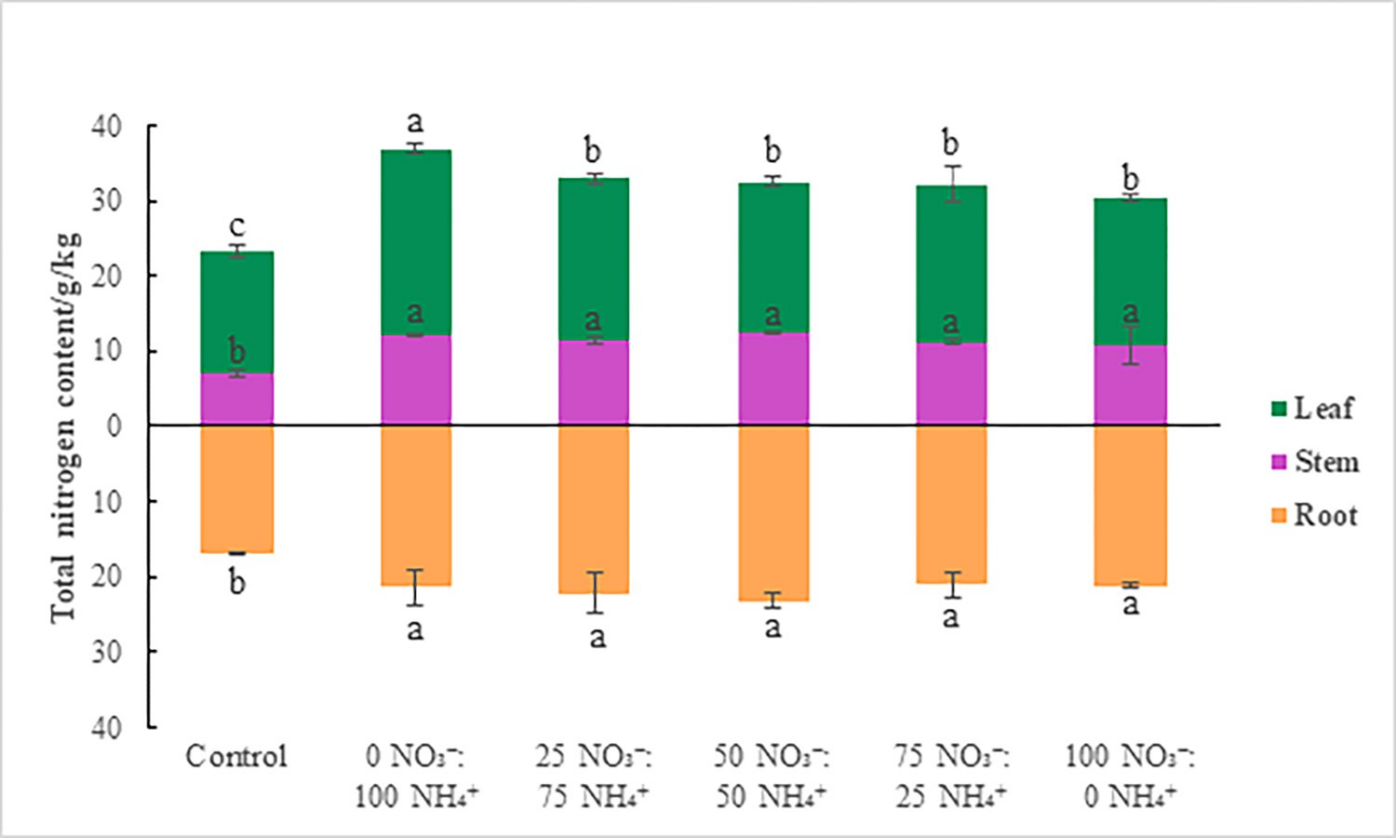
Nitrogen content in different parts of *E*. *scandens* seedlings in response to the fertilization treatments. Different lowercase letters indicate a significant difference between the treatments (*P* < 0.05).

Significant differences (*P* < 0.05) in plant nitrogen content were observed among treatments in all parts of the seedlings on day 175 (Fig 3 and S6 Table in S1 File). Total nitrogen contents of the roots, stems, and leaves were maximal in the 50 NO_3_^-^:50 NH_4_^+^, 50 NO_3_^-^:50 NH_4_^+^, and 0 NO_3_^-^:100 NH_4_^+^ treatments (20.27, 12.49, and 24.82 g/kg, respectively). Nitrogen accumulation was the highest in leaves, followed by roots and stems. As NO_3_^-^ increased, the total nitrogen content of all parts increased first and then decreased. Adding NO_3_^-^ promoted root nitrogen accumulation and the leaf nitrogen accumulation was highest under the 0% NO_3_^-^:100% NH_4_^+^ treatment.

### 3.4 Substrate pH and nitrogen content

**Fig 4:**
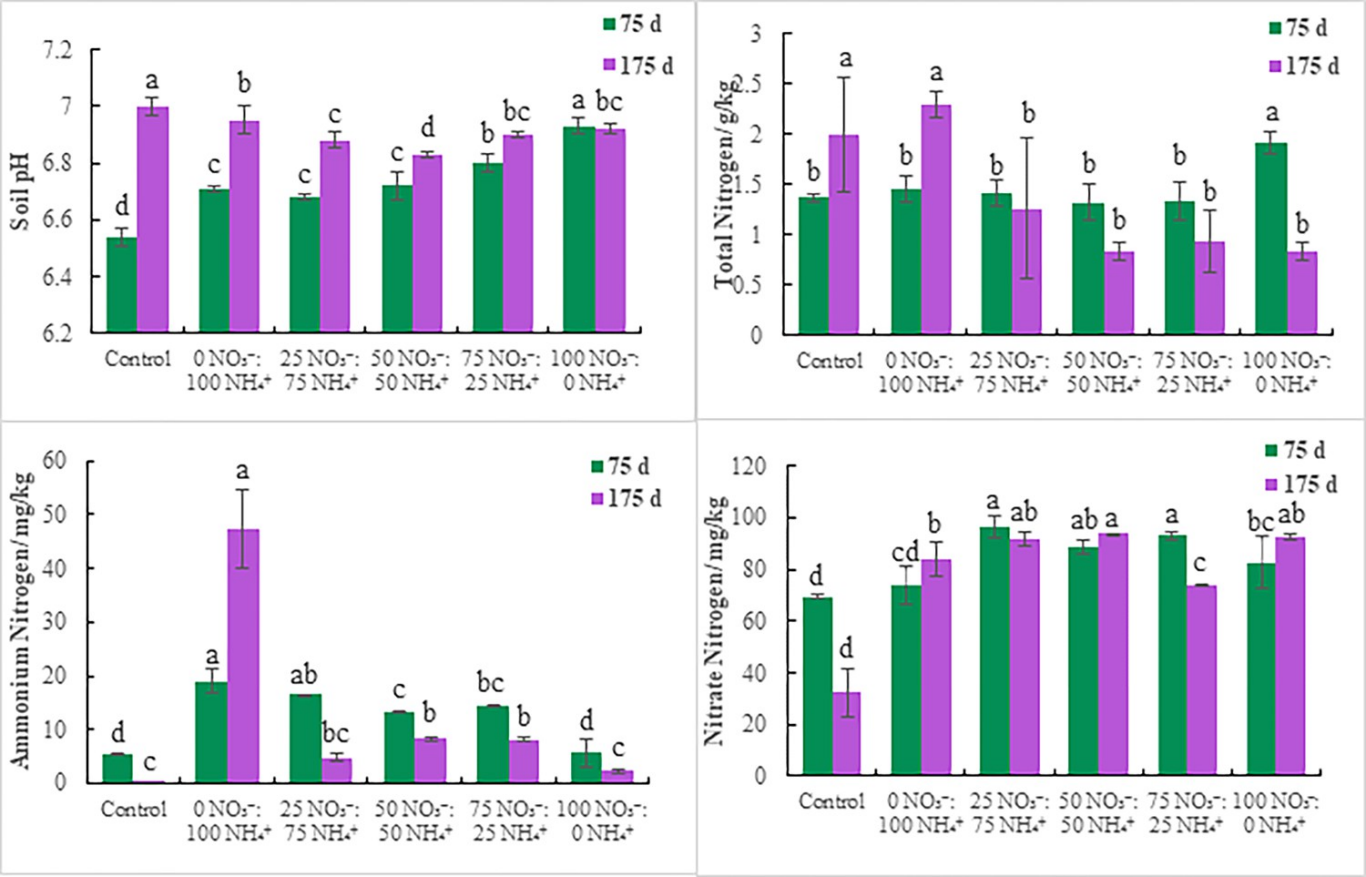
Substrate properties of *E*. *scandens* seedlings under the fertilization treatments. Different lowercase letters indicate a significant difference between the treatments (*P* < 0.05).

**1) pH:** Significant differences in substrate pH were detected among treatments on days 75 and 175 (*P* < 0.05) (Fig 4 and S7 Table in S1 File). Except for the 100 NO_3_^-^:0 NH_4_^+^ treatment, the pH values of the other treatments were higher on day 175 than day 75. The highest pH was detected in the 100 NO_3_^-^:0 NH_4_^+^ treatment (6.93) on day 75 and in the control (7.00) on day 175. As NO_3_^-^ increased the pH gradually increased on day 75, but decreased first and then increased on day 175. These results indicate that rational application of nitrogen fertilizer does not cause substrate acidification.

**2) Nitrogen content:** Significant differences in substrate total nitrogen, ammonium-nitrogen, and nitrate-nitrogen contents were observed among treatments on days 75 and 175 (*P* < 0.05). Total nitrogen content on day 175 in the control and 0 NO_3_^-^:100 NH_4_^+^ treatment was higher than on day 75, but the content was lower on day 175 in the other treatments than on day 75. Except for the 0 NO_3_^-^:100 NH_4_^+^ treatment, NH_4_^+^content on day 175 was lower than on day 75. NO_3_^-^ content was lower under the control, 25 NO_3_^-^:75 NH_4_^+^, and 75 NO_3_^-^:25 NH_4_^+^ treatments on day 175 than day 75, while the contents of the other treatments were higher than on day 75. During fertilization, total nitrogen content was highest in the 100 NO_3_^-^:0 NH_4_^+^ and 0 NO_3_^-^:100 NH_4_^+^ treatments (1.92 and 2.30 g/kg, respectively). NH_4_^+^ was highest in the 0 NO_3_^-^:100 NH_4_^+^ treatment, reaching 18.93 mg/kg and 47.39 mg/kg. In the 25 NO_3_^-^:75 NH_4_^+^ and 50 NO_3_^-^:50 NH_4_^+^ treatments, the maximum NO_3_^-^ was 96.35 and 93.82 mg/kg, respectively, indicating that the nitrogen demand of *E*. *scandens* was high. In the control, NH_4_^+^ and NO_3_^-^ decreased on day 175 compared with day 75, and NH_4_^+^ decreased more than NO_3_.

## 4 Discussion

### 4.1 *E*. *scandens* preference for different nitrogen forms

Nitrogen plays a very important role in plant growth and development. As shown by this study, plant preference for various forms of nitrogen is affected by many factors, including internal factors (plant characteristics), external factors (environmental factors), and both in combination. Although some studies have shown that ammonium toxicity can occur when excessive NH_4_^+^ is absorbed by plants [38,39], our results show that *E*. *scandens* seedlings grew well under the NH_4_^+^-N treatment. These results are similar to those of Yang et al. [40] for *Camellia sinensis* and some other species, but this is the first study to report on the tolerance of and preference for different nitrogen forms of *E*. *scandens*. Alt et al. [41] showed that blueberry prefers NH_4_^+^ to NO_3_^-^. Wang et al. [42] reported that *Camellia oleifera* prefers NH_4_^+^ to NO_3_^-^, NH_4_^+^ promotes the accumulation of amino acids in tea, particularly theanine, glutamate, and arginine, and NH_4_^+^ regulates nitrogen metabolism. Tian et al. [43] showed that the root growth of *Sophora japonica* under a total NH_4_^+^ treatment was stronger than that under a total NO_3_^-^ treatment with no NaCl, but the difference in the aboveground parts was not significant. Duan et al. [44] found that blackberry prefers to absorb NH_4_^+^, and that nitrogen assimilation and metabolism were enhanced under the NH_4_^+^ treatment, with higher biomass, Chl, antioxidant, N contents, and antioxidant enzyme activities, as well as good growth status. Similarly, a pure NH_4_^+^-N treatment enhanced the root growth and photosynthetic capacity of *Cunninghamia lanceolata* seedlings, and *Eriobotrya japonica* seedlings were most vigorous under an NH_4_^+^-N treatment [45,46]. Therefore, although NH_4_^+^ is toxic to most plants, many plants grow well under a total NH_4_^+^ treatment. Previous studies have indicated that this may be explained by the following factors.

First, NH_4_^+^ diffuses easily in soil, and plants consume less energy when they absorb NH_4_^+^ than NO_3_^-^ [47–49]. Saleh et al. [50] reported that celery (*Apium graveolens*) consumes less energy when taking up NH_4_^+^ than NO_3_^-^ because NH_4_^+^ does not have to be reduced. In addition, other studies have shown that NO_3_^-^ is better absorbed than NH_4_^+^ in deep soil with low soil nitrogen content [32]. In the current study, the root systems of the 12-month-old *E*. *scandens* seedlings had just begun to develop and fibrous roots were dominant. Therefore, the energy supply for the seedling root systems was limited; this could explain why the *E*. *scandens* seedlings preferred NH_4_^+^-N, although further study is needed to confirm this.

Second, *E*. *scandens* is mainly distributed in the southwest limestone mountainous area of Guangxi, in a karst landform area where soil pH is low [51]. These conditions limit nitrification to a certain extent. NH_4_^+^ is the main form of nitrogen in acidic red soils of southern China [52]. *E*. *scandens* prefers absorbing NH_4_^+^ due to long-term habitat selection.

Third, some studies have shown that shade-tolerant plants absorb ammonium to overcome the difficulty of NO_3_^-^ assimilation caused by low photosynthetic rates [46,53]. For example, *Panax notoginseng* survives in a shady environment with wet soil for a long time. The number of microorganisms controlling nitrification decreases in moist soil due to hypoxia, so NH_4_^+^ that accumulates in large quantities in the soil is difficult to convert into other forms [31]. As an interlayer plant in the forest [3], *E*. *scandens* is shade-tolerant. It grows in tropical rainforests and some mountainous areas, and this habitat may also have led to its preference for NH_4_^+^.

In short, these three factors may explain why *E*. *scandens* had such a strong preference for NH_4_^+^. However, the underlying mechanism of adaptation to NH_4_^+^ needs to be explored.

### 4.2 Effects of nitrogen application on *E*. *scandens* growth status

Applying nitrogen promoted the growth of *E*. *scandens* leaves and branches. No significant differences in primary root or stem biomass were observed among treatments. However, significant differences were detected in fibrous root dry weight, leaf dry weight, and total dry weight among the fertilization treatments in this experiment. The aboveground growth and biomass accumulation results were similar to those of many previous studies. Ma et al. [5,6] reported that N mainly affected the growth and biomass accumulation of *E*. *scandens*. However, no significant differences in the remaining growth indicators (root surface area or total root volume) were observed between the treatments, which differs from the results of most studies. Ma et al. [46], Huang et al. [54], and Ma et al. [55] conducted experiments on *Eriobotrya japonica*, *Beia vulgaris*, *Brassica pekinensis*, and other plants, and reported that nitrogen had a more significant effect on root growth than ammonium. Tian et al. [43] reported similar results for *Sophora japonica*. However, Chang et al. [56] found no significant difference in root surface area or the length of thick roots according to the nitrate and ammonium ratio of fertilizer for male *Populus tomentosa* seedlings. As the first part of the plant, roots may be less affected by nitrogen fertilization. Nitrogen mainly promotes the growth of leaves and aboveground parts; its growth-promoting effect on roots and stems is weaker than that of other elements, such as phosphorus and potassium. A similar pattern is seen in alfalfa, *Carthamus tinctorius*, *Cunninghamia lanceolata*, and *Pinus massoniana*. Liu et al. [57] and Hu et al. [58] showed that the root growth status of alfalfa and *C*. *tinctorius* was not significantly affected by nitrogen fertilization. No significant differences in root biomass, average root diameter, or total root length were observed under nitrate and ammonium-nitrogen treatments between *Cunninghamia lanceolata* and *Pinus massoniana* [8]. In the early stage, Ma et al. [5,6] observed differences in the nitrogen levels of 18-month-old *E*. *scandens* seedlings according to the nitrogen, phosphorus, and potassium ratios. However, whether this difference was related to the synergistic effects of nitrogen, phosphorus, and potassium remains to be explored.

### 4.3 Effects of nitrogen application on the physiological status and nitrogen content of *E*. *scandens*

A reasonable NH_4_^+^: NO_3_^-^ ratio improves the physiological status of plants. The Chl content of *E*. *scandens* was highest under the total NH_4_^+^ treatment in this study, similar to the results of Zhang et al. [59] and Chen et al. [60] for blueberry and *Carya illinoinensis*, respectively. Zhang et al. [59] showed that blueberries have the best growth and overall status under NH_4_^+^:NO_3_^-^ = 5:1 conditions, as a high NO_3_^-^ ratio inhibited photosynthesis. Chen et al. [60] showed that the ground diameter and photosynthesis of *C*. *illinoinensis* improved after a single application of NO_3_^-^, but the physiological status and nutrient accumulation were best under the total NH_4_^+^ treatment. NH_4_^+^:NO_3_^-^ = 75:25 was the optimal application ratio to prevent toxicity of a single NH_4_^+^ application. In addition, Xu et al. [61] reported that the Chl content of blueberries was highest when the ammonium: nitrate-nitrogen ratio was 2:1. Other studies have shown that enriching NH_4_^+^ in plants increases active iron content, promotes the formation of ferritin in leaves, and increases photosynthetic pigment contents [62]. Thus, these plants all prefer NH_4_^+^. Adding NH_4_^+^ greatly improves the physiological condition of plants. The soluble sugar and soluble protein contents increased after NO_3_^-^ was added, similar to the results of Hua et al. [33] and Naseri et al. [63] for *Ginkgo biloba* and *Dracocephalum moldavica*, respectively. Reasonable application of NO_3_^-^ under NH_4_^+^ conditions increases osmoregulatory substances. However, we found that, with an increase in NO_3_^-^ rate, the accumulation of proline increased, which may have resulted in mild stress; however, this stress may have been caused by excessive Ca (NO_3_) _2_. Studies have shown that excessive calcium in soil inhibits plant growth [64], and Yuan et al. [65] and Zhang et al. [66] showed that Ca (NO_3_)_2_ inhibits the growth of plants such as cucumber and tomato. However, it remains to be determined whether the performance of *E*. *scandens* in this study was attributable to calcium or excessive concentrations of Ca (NO_3_)_2_. In this study, the nitrogen contents of different tissues of *E*. *scandens* were in the order of leaves > roots > stems, and the aboveground content was higher than the underground content. This nitrogen distribution was consistent with the results of Boschiero et al. [15] for sugarcane plants, respectively, and similar to that previously reported for *E*. *scandens* [5,6]. However, when Su et al. [67] applied nitro-ammonium fertilizer to *Agastache rugosa* under different ratios, the nitrogen content varied among different plant parts, although for most plants the order was leaves > stems > roots. This may result be related to the species and other factors, although leaves are known to store high concentrations of nitrogen for photosynthesis, metabolism, and other activities.

### 4.4 Effects of applying nitrogen on the substrate status of *E*. *scandens*

The results of this study showed that the pH of the substrate decreased first and then increased as the NO_3_^-^ application ratio increased, similar to the results of Carr et al. [16] for coffee plants. Some studies have shown that applying NH_4_^+^ acidifies soil [32,68]; however, in the present study, the substrate pH was higher at day 175 than day 75, except in the total NO_3_^-^ treatment. This may be due to the preference of *E*. *scandens* for NH_4_^+^, which was quickly absorbed when applied to the substrate. The pH of the substrates in most treatments gradually increased during the fertilization and growth of the seedlings. In addition, plants release HCO_3_^−^ into the soil after absorbing NO^-^_3_, thus increasing soil pH [17]. These two aspects were responsible for the change in substrate pH observed in this study. In addition, the total nitrogen and NH_4_^+^ contents in most of the treatments trended downward after fertilization, as nitrogen was absorbed by *E*. *scandens*. Compared with NO_3_^-^, *E*. *scandens* preferred to absorb NH_4_^+^, which resulted in a significant reduction in NH_4_^+^ under all treatments except the total NH_4_^+^ treatment. Overall, the NH_4_^+^ nitrogen content decreased in all treatments except the total NH_4_^+^ nitrogen treatment, and NH_4_^+^ was the main form of nitrogen absorbed and utilized by *E*. *scandens*.

## 5 Conclusion

In this study, the growth, physiological characteristics, and nitrogen content of 12-month-old *E*. *scandens* seedlings were investigated under different NH_4_^+^ and NO_3_^-^ fertilization ratios. The results showed that the demand for nitrogen was very strong. A reasonable nitro-ammonium fertilization ratio promoted the growth of aboveground leaves and branches, but the effect of the nitro-ammonium fertilization ratio on root growth was not significant. Plant growth was slightly inhibited by the increase in nitrate content. Among all treatments, the 0 NO_3_^-^:100 NH_4_^+^ treatment (ammonium chloride 3.82 g/plant) had the best effect on the growth and physiological status of *E*. *scandens*.

## Supporting information

S1 File Supporting information contains S1-S7 Tables. (DOCX)
