# Supplementary material for: Effects of the nitrate and ammonium ratio on plant characteristics and Erythropalum scandens Bl. substrates
Source: PLoS One. 2023 Aug 4;18(8):e0289659. doi: 10.1371/journal.pone.0289659 (PMC10403090; doi:10.1371/journal.pone.0289659)
Supplement: S1 File — (DOCX) [file pone.0289659.s001.docx]

# Supporting information

**S1 Table F-value and P-value of aboveground growth indexes**

| Index | Number of  new buds | Number of  new leaves | Number of  new branches | Number of  basal branches | Sum of  branches |
| --- | --- | --- | --- | --- | --- |
| F-value | 0.985 | 3.964 | 1.863 | 1.381 | 2.534 |
| P-value | 0.431 | 0.003 | 0.107 | 0.238 | 0.033 |
| Index | Total length of  new branches | Number of  new internodes | Mean length  of internode | Ground diameter  increment | New branch  thickness |
| F-value | 6.156 | 3.741 | 3.489 | 2.167 | 2.57 |
| P-value | 0 | 0.004 | 0.006 | 0.063 | 0.031 |

| **S2 Table F-value and P-value of underground growth indexes** | | | | | |
| --- | --- | --- | --- | --- | --- |
| Index | Total root  length | Total root  surface area | Total root  projection area | Total root  volume | Mean root  diameter |
| F-value | 4.049 | 0.933 | 0.933 | 0.994 | 2.936 |
| P-value | 0.022 | 0.494 | 0.493 | 0.461 | 0.059 |

**S3 Table F-value and P-value of biomass**

| Index | Main root  biomass | Fibrous root  biomass | Root  biomass | Stem  biomass | Leaf  biomass | Total  biomass |
| --- | --- | --- | --- | --- | --- | --- |
| F-value | 0.504 | 3.758 | 1.638 | 1.789 | 7.268 | 4.366 |
| P-value | 0.768 | 0.028 | 0.224 | 0.19 | 0.002 | 0.017 |

| **S4 Table F-value and P-value of chlorophyll content** | | | | | | | | |
| --- | --- | --- | --- | --- | --- | --- | --- | --- |
| Index | Chlorophyll a | |  | Chlorophyll b | |  | Chlorophyll a+b | |
|  | 75 d | 175 d |  | 75 d | 175 d |  | 75 d | 175 d |
| F-value | 4.165 | 9.864 |  | 7.565 | 2.207 |  | 5.803 | 5.469 |
| P-value | 0.02 | 0.001 |  | 0.002 | 0.121 |  | 0.006 | 0.007 |

| **S5 Table F-value and P-value of osmotic regulators content** | | | | | | | | |
| --- | --- | --- | --- | --- | --- | --- | --- | --- |
| Index | Soluble sugar content | |  | Soluble protein content | |  | Free proline content | |
|  | 75 d | 175 d |  | 75 d | 175 d |  | 75 d | 175 d |
| F-value | 1.46 | 0.917 |  | 6.869 | 3.5 |  | 0.568 | 3.684 |
| P-value | 0.273 | 0.502 |  | 0.003 | 0.035 |  | 0.723 | 0.03 |

**S6 Table F-value and P-value of plant nitrogen content**

| Index | Root total  nitrogen content | Stem total  nitrogen content | Leaf total  nitrogen content |
| --- | --- | --- | --- |
| F-value | 5.044 | 9.453 | 17.804 |
| P-value | 0.01 | 0.001 | 0 |

| **S7 Table F-value and P-value of substrate properties** | | | | | | | | | | | |
| --- | --- | --- | --- | --- | --- | --- | --- | --- | --- | --- | --- |
| Index | Substrate pH | |  | Substrate total  nitrogen content | |  | Substrate ammonium  nitrogen content | |  | Substrate nitrate  nitrogen content | |
|  | 75 d | 175 d |  | 75 d | 175 d |  | 75 d | 175 d |  | 75 d | 175 d |
| F-value | 60.612 | 13.098 |  | 7.854 | 7.777 |  | 47.353 | 101.54 |  | 11.17 | 72.4 |
| P-value | 0 | 0 |  | 0.002 | 0.002 |  | 0 | 0 |  | 0 | 0 |
